# Impact of a Novel Antiseptic Lavage Solution on Acute Periprosthetic Joint Infection in Hip and Knee Arthroplasty

**DOI:** 10.3390/jcm13113092

**Published:** 2024-05-24

**Authors:** Luca Andriollo, Rudy Sangaletti, Calogero Velluto, Loris Perticarini, Francesco Benazzo, Stefano Marco Paolo Rossi

**Affiliations:** 1 Robotic Prosthetic Surgery Unit—Sports Traumatology Unit, Fondazione Poliambulanza Hospital, 25124 Brescia, Italyfrancesco.benazzo@poliambulanza.it (F.B.); 2 Department of Orthopedics, Catholic University of the Sacred Heart, 00168 Rome, Italy; 3 Biomedical Sciences Area, IUSS University School for Advanced Studies, 27100 Pavia, Italy

**Keywords:** Bactisure Wound Lavage, PJI, joint replacement, acute periprosthetic joint infection, DAIR, DAPRI

## Abstract

**Background:** Periprosthetic joint infection (PJI) represents a challenge following hip or knee arthroplasty, demanding immediate intervention to prevent implant failure and systemic issues. Bacterial biofilm development on orthopedic devices worsens PJI severity, resulting in recurrent hospitalizations and significant economic burdens. The objective of this retrospective cohort study is to evaluate the efficacy of this novel antiseptic solution, never previously evaluated in vivo, in managing early post-operative or acute hematogenous PJI following primary hip and knee joint replacements. **Methods:** The inclusion criteria consist of patients with total hip arthroplasty (THA) or knee arthroplasty diagnosed with acute PJI through preoperative and intraoperative investigations, in accordance with the MSIS ICM 2018 criteria. The minimum required follow-up was 12 months from the cessation of antibiotic therapy. This novel antiseptic lavage solution is composed of ethanol, acetic acid, sodium acetate, benzalkonium chloride and water. Data included demographic characteristics, diagnostic criteria, surgical techniques, post-operative treatment and follow-up outcomes. **Results:** A total of 39 patients treated with Debridement, Antibiotics Pearls and Retention of the Implant (DAPRI) procedures using this solution between May 2021 and April 2023 were analyzed. At a mean follow-up of 24.6 ± 6.4 months, infection recurrence-free survival rates were 87.2%, with no local allergic reactions or relevant systemic adverse effects detected. Persistent PJI necessitated two-stage revision surgery. **Conclusions:** This novel antiseptic lavage solution shows promise as an adjunctive tool in the treatment of PJI, demonstrating support in infection control while maintaining a favorable safety profile.

## 1. Introduction

Periprosthetic joint infection (PJI) is a devastating complication of hip or knee arthroplasty, with a mean rate of approximately 1–2% [1,2]. This requires prompt and effective management to prevent implant failure and systemic effects [3,4]. PJI often necessitates repeated hospitalizations and invasive treatments, carrying a considerable risk of significant adverse events. As a consequence, the total cost associated with treating PJI ranks among the highest for orthopedic procedures [5,6,7,8].

A pivotal aspect in the development of PJI is the production of biofilm by bacteria on the surfaces of implanted orthopedic devices. Biofilm is an extracellular-produced polymeric matrix that shields bacteria from the host’s immune response, antibiotic treatment and even mechanical debridement [9,10]. The maturity of biofilm is recognized as a crucial factor in distinguishing between acute and chronic PJI: An acute PJI typically involves an immature biofilm, which may be susceptible to treatment through Debridement, Antibiotics and Implant Retention (DAIR). On the other hand, chronic PJI is characterized by a mature biofilm that is often resistant to complete removal via a DAIR procedure, necessitating the complete removal of the implanted components [11,12].

DAIR, and its evolution, Debridement, Antibiotic Pearls and Retention of the Implant (DAPRI) procedures, are reliable strategies for addressing acute PJIs. Even though the DAIR/DAPRI procedure is quite limited, it remains a viable option for patients developing early post-operative or acute hematogenous PJI, with success rates ranging from 55.5% to a maximum of 90% [12,13]. However, the impact on the effectiveness of these interventions of the lavage solutions used during the surgical procedure is not completely clear.

The intraoperative irrigation solution serves as an additional tool for surgeons to address intraoperative contamination and minimize bacterial load. Typically, this solution is either an antibiotic or a diluted antiseptic applied to the surgical wound after mechanical debridement of affected periarticular tissue [14].

In the literature, there are several studies evaluating the effectiveness of antiseptic solutions available on the market through in vitro studies [15,16,17,18]. However, clinical studies assessing their efficacy are lacking, particularly for Bactisure^®^ Wound Lavage (Zimmer Biomet, Warsaw, IN, USA), a novel antiseptic solution composed of ethanol, acetic acid, sodium acetate, benzalkonium chloride and water, which has not yet been evaluated in vivo.

The aim of this study is to assess the impact of applying Bactisure^®^ Wound Lavage in the treatment of early post-operative or acute hematogenous PJI following primary hip and knee joint replacements. Specifically, the study aims to evaluate the rate of infection recurrence-free survival at the final follow-up and analyze any adverse effects observed during its clinical application.

The hypothesis is that Bactisure^®^ Wound Lavage could be a useful tool in the management of PJIs, safely usable in DAIR/DAPRI procedures.

## 2. Materials and Methods

Patients diagnosed with PJI on primary hip or knee prostheses treated with DAPRI and the use of Bactisure^®^ Wound Lavage (Zimmer Biomet, Warsaw, IN, USA), a novel antiseptic solution, were retrospectively evaluated. The patients included in the study were treated between May 2021 and April 2023. All procedures were conducted at a single high-volume center for primary and revision prosthetic surgery.

The inclusion criteria consist of patients with total hip arthroplasty (THA) or knee arthroplasty, both unicompartmental (UKA) and total (TKA), diagnosed with acute PJI through preoperative and intraoperative investigations, in accordance with the MSIS ICM 2018 criteria [19]. For acute infections where the implant is stable and there is sufficient soft tissue mass, recent guidelines suggest retaining the implant for treating PJI occurring within 30 days after arthroplasty or with symptoms present for less than 3 weeks, including acute hematogenous PJI [2].

The minimum required follow-up for inclusion in this study was 12 months from the cessation of antibiotic therapy. 

Exclusion criteria included: age < 18 years; incomplete pre-operative, intra-operative or follow-up data; history of post-traumatic osteoarthritis; previous surgical interventions on the joint treated with arthroplasty; PJI in the setting of revision joint arthroplasty; history of alcohol abuse or drug addiction; or other patient characteristics indicative of poor compliance with the treatment path.

Demographic data of patients were collected, including historical risk factors such as diabetes, active smoking, rheumatoid arthritis and immunodeficiency conditions. Detailed data on the primary surgery were accurately recorded for all patients, including the type of prosthetic treatment and the duration of the procedure itself. For early post-operative infections (within 30 days after arthroplasty), the time from the primary surgery to the DAPRI procedure was reported, while for acute hematogenous PJI (with symptoms present for less than 3 weeks), the days from symptom onset, or from the episode of hematogenous infection if known, to treatment with DAPRI were documented.

Furthermore, clinical and laboratory data used for the diagnosis of PJI were reported in accordance with the MSIS ICM 2018 criteria. The preoperative alpha-defensin test was performed using Synovasure^®^ (Zimmer Biomet, Warsaw, IN, USA). Throughout all surgical procedures, a frozen section was addressed with a histopathological analysis; it was considered positive when greater than 5 neutrophils per high-power field in 5 high-power fields were observed via a histological analysis of periprosthetic tissue at 400× magnification. Additionally, the presence of intra-articular purulence was reported during the surgical procedure for the treatment of PJI. These were crucial in cases of “Possibly infected” for definitive intraoperative confirmation of infection. Intraoperative cultures were performed in all patients, with results reported and evaluated.

All patients underwent consultation with an infectious disease specialist, with tailored antibiotic therapy prescribed. Serial hematologic tests were conducted during follow-up.

Local and systemic adverse reactions to Bactisure^®^ were evaluated. Specifically, data from medical records were analyzed for any local allergic or inflammatory reactions, as well as for pulmonary, cardiac, neurological or renal function alterations.

Infection at the final follow-up was considered eradicated in the absence of clinical signs of PJI and with hematologic tests showing negative inflammatory and infectious markers for at least 12 months after cessation of antibiotic therapy.

### 2.1. Diagnostic and Therapeutic Protocol in the Management of Acute PJI

For every potential case eligible for treatment with DAPRI, a thorough array of assessments is carried out. This comprises blood analyses covering a complete blood count, Erythrocyte Sedimentation Rate (ESR) and C-Reactive Protein (CRP), along with targeted X-ray imaging of the affected joint, as well as arthrocentesis guided by ultrasound.

**Figure 1:**
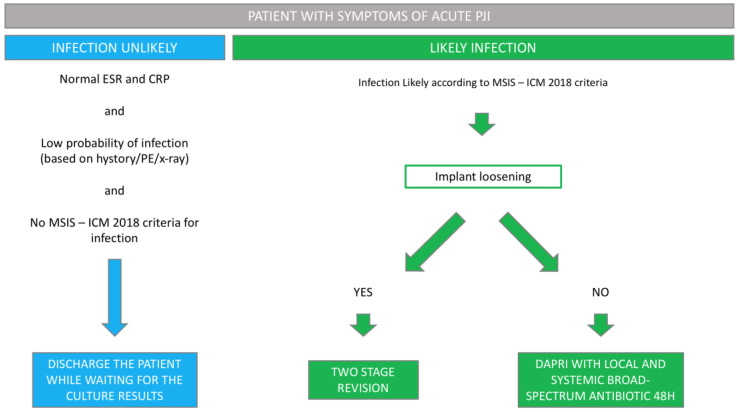
Diagnostic and Therapeutic Protocol in the Management of Acute Periprosthetic Joint Infection (PJI) (Erythrocyte Sedimentation Rate, ESR; C-Reactive Protein, CRP; physical examination, PE; Debridement, Antibiotic Pearls and Retention of the Implant, DAPRI).

In patients with suspected acute symptoms of PJI, following the application of the MSIS ICM 2018 criteria, the diagnostic and therapeutic algorithm outlined in Figure 1 was implemented.

Patients with normal ESR and CRP levels, a low probability of infection based on history, physical examination and X-rays, and absence of positivity for infection according to the MSIS ICM 2018 criteria were categorized as “Infection unlikely.” For this group of patients, the recommendation was discharge while awaiting culture results of synovial fluid obtained through arthrocentesis.

In patients with positive MSIS ICM 2018 criteria, surgical intervention was indicated. In the absence of implant loosening, patients falling into this “Likely infection” group with early post-operative infections (within 30 days after arthroplasty) or acute hematogenous PJI (with symptoms present for less than 3 weeks) underwent a DAPRI procedure.

### 2.2. Bactisure^®^ Wound Lavage: Characteristics and Mode of Use

Bactisure^®^ Wound Lavage is a medical solution designed for the removal of debris, including microorganisms, from wounds through pulsed (jet) lavage. This product is a clear, colorless and low-odor solution, meeting the standards set by the FDA as a 510(K) cleared device. It contains active ingredients such as ethanol, acetic acid, sodium acetate, benzalkonium chloride and water. Available in sterile 1000 mL polypropylene plastic bags equipped with an integrated single spike port, Bactisure^®^ is meticulously formulated for optimal wound care.

**Figure 2:**
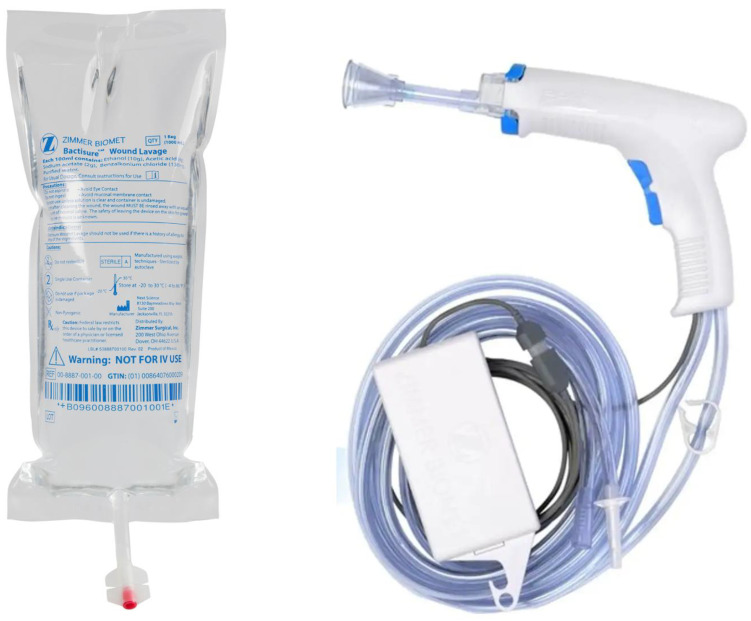
Bactisure^®^ Wound Lavage (Zimmer Biomet, Warsaw, IN, USA) and Pulsavac^®^ Plus (Zimmer Biomet, Warsaw, IN, USA), a pulsatile lavage system required for its application.

The indications for its use cover all wound types, making it a versatile tool in clinical settings. It is recommended to apply Bactisure^®^ Wound Lavage just prior to wound closure, preferably using a Zimmer Biomet Pulsavac^®^ Plus (Zimmer Biomet, Warsaw, IN, USA), a similar pulsed lavage system (Figure 2). 

Following application, the wound should be immediately rinsed with an equal amount of normal saline using pulsed lavage. However, it is important to note that Bactisure^®^ Wound Lavage is not intended for repeated use or during dressing changes. It should not be soaked into dressings either, to ensure its effectiveness and avoid unnecessary complications [18]. 

Individuals with a history of allergy to any of the ingredients in Bactisure^®^ Wound Lavage should avoid its use. However, for those without such contraindications, the solution has been demonstrated to be safe for human tissue. Multiple tests and reports, including NAMSA Toxicology Reports and ISO Intramuscular Implantation Tests with Histopathology, have confirmed its non-irritant nature and its promotion of normal wound healing [14,20,21].

One of the key features of Bactisure^®^ Wound Lavage is its ability to break up crosslinks within biofilm Extracellular Polymeric Substance (EPS), effectively deconstructing biofilms. By solubilizing biofilms for easy removal via pulsed lavage, Bactisure^®^ aids in the eradication of persistent wound infections. This is particularly significant given the role of biofilms in chronic wound infections, where over 90% of all bacteria are believed to exist. Biofilms are formed when bacteria coalesce on surface structures and produce EPS, which shields them from both mechanical and chemical attack, rendering them significantly more resistant to antibiotics than free-floating bacteria. Bactisure^®^’s ability to disrupt biofilms is therefore crucial in enhancing wound care practices and combating infections [15,17,22].

In terms of efficacy, independent laboratory testing has shown that Bactisure^®^ Wound Lavage effectively removes common wound pathogens, including bacteria found in biofilms. These findings underscore its potential to enhance standard wound care practices and infection control measures when used as an adjunct to normal saline wound lavage [17,23].

The rationale for using Bactisure^®^ Wound Lavage in acute PJI is twofold: it serves both as a treatment for soft tissues and wound management, and as an action against the initial formation of biofilm on inert prosthetic material, which can be difficult to completely remove. Infectious residue on prosthetic components can indeed act as a reservoir for recurrence. 

### 2.3. Surgical Technique for DAPRI with Bactisure^®^ Wound Lavage

In acute PJI, defined as occurring within 30 days after arthroplasty or with symptoms present for less than 3 weeks, including acute hematogenous PJI, DAPRI procedures were performed [2]. Even in the absence of positive synovial fluid culture results, broad-spectrum local antibiotics were applied at the time of surgery.

The surgical approach for both knee and hip joints was based on the original technique described by Indelli, with some variations outlined here [24,25]

The DAPRI procedure has a stepwise approach. Preoperative antibiotic therapy is intentionally held to improve the sensitivity of intraoperative cultures.

#### 2.3.1. Knee PJI

The patient is positioned supine. Prior to skin incision and arthrotomy, a large needle is inserted into the knee joint to aspirate as much fluid as possible, which is then sent for culture. Following this, a solution of dilute 0.1% methylene blue (made by mixing 40 mL of normal saline with 10 mL of 0.5% methylene blue solution) is injected into the knee joint, following Shaw’s technique [26]. Methylene blue is used because it stains bacterial biofilm. Afterward, a sterile arthrocentesis is performed to remove as much dye as possible from the joint before arthrotomy. 

A standard medial parapatellar approach and capsulotomy are carried out. Immediately after the arthrotomy, suction is used to remove any remaining dye from the joint. Five tissue samples are taken from different areas within the joint and stained for microbiological analysis (aerobic, anaerobic and fungal exams). A sample of periarticular tissue was sent to the laboratory for frozen section with histopathological analysis. The polyethylene liner is then removed.

With wide exposure, an extensive and radical synovectomy is performed, including the synovial layer on the posterior capsule, aiming to eliminate all stained soft tissue.

Subsequently, a brush containing 4% chlorhexidine gluconate is used to scrub all visible surfaces of the femoral, tibial and patellar components. This mechanical action is performed to remove biofilm.

Finally, pulse irrigation with 6 L of NaCl supplemented with 4% povidone-iodine is performed. At this point, the surgical team discards their used gowns and gloves, and the back table with contaminated instruments is no longer utilized. A new sterile drape, supplemented with povidone-iodine, is applied to the skin, and the surgical team dons new gowns and gloves, while a new back table with sterile instruments is prepared for use.

Subsequently, the final lavage is carried out with 1 L of Bactisure^®^ Wound Lavage using low-intensity pulse irrigation, followed by 1 L of NaCl. The new liner is then positioned. 

As a last step before hermetically suturing the joint capsule, a 10 mL kit of PG-CSH (Stimulan^®^, Biocomposites Ltd., Keele, UK) supplemented with 1000 mg of vancomycin hydrochloride powder and 240 mg of liquid Gentamicin solution is applied as broad-spectrum antibiotic therapy in the absence of culture results. Alternatively, if an antibiogram is available, targeted antibiotic therapy is administered. Stimulan^®^, after adequate preparation, is applied in the form of calcium sulfate beads.

#### 2.3.2. Hip PJI

The DAPRI surgical procedure applied to the hip followed the same steps as described for the knee. Specifically, the patient was positioned on the contralateral side, with a postero-lateral surgical approach retracing the previous surgical incision (all treated patients had received a primary implant via the postero-lateral approach).

After adequate joint exposure achieved through prosthetic dislocation, the femoral head and acetabular liner were removed. The femur was then positioned superiorly and anteriorly. Five tissue samples were taken for culture examination, and a sample of periarticular tissue was sent to the laboratory for frozen section with histopathological analysis.

An aggressive and radical “tumor-like” synovectomy and capsulotomy, including the posterior capsule layer, were then performed with the aim of removing all soft tissues contaminated by the infected biofilm. Subsequent steps included scrubbing with a brush containing 4% chlorhexidine gluconate, pulse irrigation with 6 L of NaCl supplemented with 4% povidone-iodine and changing of sterile drapes, gowns, gloves and surgical instruments.

The final lavage is carried out with 1 L of Bactisure^®^ Wound Lavage using low-intensity pulse irrigation, followed by 1 L of NaCl. The new liner is then positioned. 

As a final step, Stimulan^®^ (Biocomposites Ltd., UK) was applied using the same methods described previously. In this case as well, hermetic closure of the fascia is essential.

### 2.4. Post-Operative Treatment and Follow-Up

All patients undergo a standardized postoperative rehabilitation protocol, which includes immediate joint mobilization and weight-bearing as tolerated with crutches on the day of surgery. Postoperative antibiotic treatment is routinely initiated based on preoperative and intraoperative findings in consultation with our institutional infectious disease service. Following a DAPRI procedure, patients typically receive intravenous and/or oral antibiotic therapy for a duration ranging from 6 to 12 weeks. 

During antibiotic treatment, patients undergo hematological examinations every 14 days, including complete blood count, ESR and CRP levels. After suspension of antibiotic therapy, patients undergo regular examinations for up to 12 months post-therapy cessation. Additionally, patients are periodically evaluated during follow-up appointments to monitor for any clinical signs of potential infectious recurrence.

### 2.5. Statistical Analysis

Statistical analysis was performed using SPSS v18.0 (Chicago, IL, USA) by an independent statistician. Continuous variables were reported using average and standard deviation (SD), while categorical variables were presented using frequency distributions and percentages.

Level of Evidence III: retrospective cohort study.

## 3. Results

From May 2021 to April 2023, 39 patients diagnosed with acute PJI underwent treatment with DAPRI using Bactisure^®^ Wound Lavage (Zimmer Biomet, Warsaw, IN, USA). Among these patients, 23 were male (59%) and 16 were female (41%). The average age at the time of surgery was 65.9 ± 11.8 years. The surgical side was left in 27 cases (69.2%) and right in 12 cases (30.8%). Of these patients, 31 (79.5%) were treated for early post-operative PJI (within 30 days after arthroplasty), while 8 (20.5%) were treated for acute hematogenous PJI (with symptoms present for less than 3 weeks). The primary arthroplasty intervention involved 11 patients (28.2%) undergoing THA, 21 patients (53.8%) undergoing TKA and 7 patients (18%) undergoing UKA. 

Baseline demographics are detailed in Table 1.

In patients treated for early post-operative PJI, the mean time from primary replacement to DAPRI treatment was 19.7 ± 6.7 days. In patients with acute hematogenous PJI, the mean time from symptom onset to DAPRI treatment was 13.6 ± 5.7 days.

Table 2 presents the data of parameters for the diagnosis of PJI according to the MSIS ICM 2018 criteria.

According to the MSIS ICM 2018 criteria, 36 patients (92.3%) were classified as “Infected” based on preoperative examinations, while the remaining 3 patients (7.7%) who initially had a “Possibly infected” score preoperatively were subsequently confirmed to have acute PJI through intraoperative examinations.

Table 3 contains the data on intraoperative criteria for the diagnosis of PJI according to the MSIS ICM 2018 and surgical time.

Among the 37 patients with positive cultures, 33 (89.2%) had the isolation of a single pathogen. In 13 cases (35.1%), Methicillin-Susceptible Staphylococcus Aureus (MSSA) was isolated, in 3 cases (8.1%) Methicillin-resistant Staphylococcus Aureus (MRSA), in 10 cases (27%) Methicillin-Susceptible Staphylococcus Epidermidis (MSSE) and in 1 case (2.7%) Methicillin-Resistant Staphylococcus Epidermidis (MRSE). Other isolated pathogens included *E. coli* (2 cases), *E. faecalis* (2), *Serratia liquefaciens* (2), *Staphylococcus lugdunensis* (3), *Streptococcus sanguinis* (1), *Propionibacterium acnes* (1), *Kokuria* (1) and *Moraxella catarrhalis* (1).

In the postoperative period, no local adverse reactions of allergic or inflammatory nature were detected, despite the challenging assessment of synovial response due to concurrent use of Stimulan^®^. As for systemic adverse reactions, no alterations in pulmonary, cardiac or neurological function were reported. Two patients (5.1%) experienced moderate renal dysfunction, which normalized upon discontinuation of NSAID therapy and adjustment of analgesic treatment.

At a final follow-up of 24.6 ± 6.4 months, according to the criteria described earlier, 34 patients were considered cured of PJI (87.2%). The 5 patients with persistent PJI required a two-stage revision surgery. 

## 4. Discussion

The management of PJIs following hip and knee arthroplasty is a current issue in orthopedic surgery. PJIs are challenging complications with potentially devastating consequences, necessitating prompt and effective intervention to mitigate implant failure and systemic repercussions. Biofilm formation by bacteria on orthopedic device surfaces plays a pivotal role as a key factor in the pathogenesis of PJIs [9]. The rationale for prompt treatment is fundamental to prevent devastating consequences. For this reason, several techniques have been reported in the literature aiming to prevent worsened infections and their spread. Debridement, Antibiotics and Retention of the Implant (DAIR) were first reported by Gristina et al. [27]. The rationale behind this treatment is to remove the biofilm from the implant, which is well-known to increase infection spreading and resistance to antibiotic treatment. However, the choice of adjunctive therapies, such as adding a disinfectant solution, remains controversial, as no consensus on which one yields better clinical outcomes has been established. Several studies have attempted to remove biofilm in vitro, but clinical studies assessing their efficacy are not reported in the literature [17,23].

The integration of advanced diagnostic and therapeutic modalities within a specific protocol could represent a transformative advancement in the management of acute PJI. By aligning with established diagnostic criteria and treatment protocols, the aim is to optimize patient care and outcomes while addressing the challenges posed by this complex clinical entity. For this reason, this study reports the use of Bactisure^®^ Wound Lavage, a novel antiseptic solution, added to DAPRI, in the treatment of early post-operative or acute hematogenous PJIs. This fills a notable gap in the literature, as clinical studies assessing the effectiveness of antiseptic solutions in PJI management are limited. By retrospectively analyzing patients treated with Debridement, Antibiotic Pearls and Retention of the Implant (DAPRI) alongside Bactisure^®^ Wound Lavage, the study provides valuable insights into a potentially promising adjunctive therapy.

The success rate of DAPRI in this cohort, with an 87.2% rate of patients without infection at final follow-up, underscores the importance of precise surgical intervention and patient selection. These findings corroborate previous studies highlighting the efficacy of DAPRI in addressing acute PJIs [28,29].

Regarding the definition of acute PJI, this study emphasizes the significance of timely intervention and accurate classification of infection types. As delineated by Tsukayama et al. and Zimmerli et al., distinguishing between acute and chronic infections based on symptom duration is crucial for tailoring treatment approaches and predicting outcomes [9,30]. By aligning with these established criteria, this study proposes an extension of the treatment window for implementing the Debridement, Antibiotics and Implant Retention (DAIR) protocol, providing a nuanced strategy for addressing the evolving setting of PJI pathophysiology [24,25].

This extension is predicated on the premise that early intervention, particularly within the acute phase, offers a higher likelihood of treatment success, as supported by the findings of Youssef et al. [31]. Moreover, this study underscores the importance of integrating diagnostic criteria and treatment protocols to optimize patient care, as evidenced by the work of Parvizi et al. and Tande et al. [19,32].

Furthermore, this study introduces a comprehensive set of diagnostic protocols, integrating a multitude of investigations ranging from serological tests to advanced imaging modalities. By incorporating these diagnostic modalities into the treatment algorithm, the present study aims to enhance the accuracy of PJI diagnosis and facilitate targeted therapeutic interventions. This approach is in line with the recommendations of the Musculoskeletal Infection Society (MSIS) and the Infectious Diseases Society, which support a multidisciplinary approach to PJI management [2]. By leveraging advanced imaging techniques such as MRI and CT scans, the protocol presented in this study aims to provide detailed insights into soft tissue changes, fluid collections and bone-related issues around the joint prosthesis, facilitating early detection and intervention. Additionally, histological analysis of periprosthetic tissues enables the identification of infection signs such as inflammatory cells or bacterial biofilms, further informing treatment decisions [3,10].

Another crucial point is defining what is considered acute, because in literature there is no consensus on the timing of infection. This study sheds light on the evolving definition of acute PJI, incorporating cases with symptoms present for less than 3 weeks, aligning with emerging trends in the literature [19].

The introduction of Bactisure^®^ Wound Lavage as an adjunctive therapy is particularly intriguing, given its potential to disrupt biofilms and aid in wound management. 

Irrigation and debridement play a crucial role in reducing bioburden and subsequently lowering reinfection rates in PJI treatment. However, comparing different irrigation solutions remains challenging due to variations in treatment protocols. While in vitro studies provide valuable insights for selecting irrigation fluids, their results may not directly translate into clinical significance. 

Multiple protocols for the surgical treatment of PJI have included povidone-iodine. Studies by Zubko and Zubko demonstrated that while hydrogen peroxide and povidone-iodine are bacteriostatic when used separately, they become bactericidal when used together [33]. Normal saline is the most commonly used irrigation solution for debridement. Some studies have shown that diluted povidone-iodine is significantly more effective than saline solution in preventing PJI [34]. Nevertheless, the optimal dilution of povidone-iodine has yet to be established, with lower concentrations proving effective and causing minimal damage to host tissue [35]. Hydrogen peroxide is effective against bacteria, particularly gram-positive organisms, and does not compromise the strength of bone cement or metal implants. It also reduces bacterial biofilms. However, its use can increase the risk of air embolism [36]. 

Future research should focus on standardizing treatment protocols and conducting multicenter in vivo studies to better evaluate the clinical significance of various lavage solutions in PJI management. This approach will help in determining the most effective and safe irrigation solutions, ultimately improving patient outcomes [37]

While in vivo studies assessing antiseptic solutions are limited, in vitro evidence suggests the efficacy of such interventions in mitigating biofilm formation [14,15].

The study’s exploration of Bactisure^®^ Wound Lavage’s ability to solubilize biofilms is noteworthy, aligning with previous research demonstrating the importance of biofilm disruption in PJI management [18,22]. In addition to evaluating the efficacy of Bactisure^®^ Wound Lavage, this study delineates the diagnostic and therapeutic protocol employed in managing acute PJIs. By adhering to the MSIS ICM 2018 criteria and utilizing a comprehensive array of preoperative and intraoperative assessments, the study ensures standardized diagnostic pathways and enhances the accuracy of PJI diagnosis [19]. This aligns with current trends in orthopedic literature, emphasizing the importance of standardized diagnostic criteria in optimizing PJI management [23]. Moreover, the study’s surgical technique for DAPRI procedures provides valuable insights into the approach required to address acute PJIs effectively. The stepwise protocol, encompassing joint-specific approaches for knee and hip PJIs, underscores the importance of thorough debridement, synovectomy and biofilm disruption in achieving infection eradication. The incorporation of broad-spectrum local antibiotics and targeted antibiotic therapy based on intraoperative culture results further highlights the study’s commitment to individualized patient care [11].

The post-operative treatment and follow-up protocol described in the study offers valuable guidance for optimizing patient outcomes following DAPRI procedures. The standardized rehabilitation protocol, coupled with rigorous hematological monitoring and regular clinical assessments, ensures comprehensive post-operative care and facilitates early detection of potential infectious recurrences. These recommendations align with previous literature advocating for multidisciplinary post-operative management approaches to enhance patient recovery and minimize the risk of recurrent infections [2,4].

### Limitations

However, several considerations deserve further discussion. The absence of local adverse reactions to Bactisure^®^ Wound Lavage is encouraging, yet the potential for systemic adverse effects remains a concern. Future investigations could delve deeper into the long-term safety profile of Bactisure^®^ and explore alternative strategies to mitigate adverse reactions. The study’s limitations, including the retrospective design and lack of a control group, underscore the need for further prospective, controlled trials to validate the efficacy of Bactisure^®^ Wound Lavage in PJI management. Additionally, the exclusion of chronic PJI cases limits the generalizability of the findings, prompting future studies to address this gap in knowledge. Further research is warranted to elucidate the long-term safety and efficacy of this intervention, ultimately optimizing outcomes for patients undergoing hip and knee arthroplasty.

## 5. Conclusions

Based on these findings, it can be concluded that the results of the DAPRI protocol using Bactisure^®^ Wound Lavage are encouraging. However, without a control group, it remains uncertain whether the positive outcomes can be attributed solely to Bactisure^®^ Wound Lavage. Nonetheless, there were no reported clinical toxic results or allergic reactions associated with its use. Therefore, in the management of acute PJI, it is advisable to leverage all available resources, even with a minimal increase in cost, given the potentially devastating impact on both the patient and the cost of revising to a two-stage implant. 

In conclusion, this novel antiseptic lavage solution shows promise as an adjunctive tool in the treatment of PJI.

## Figures and Tables

**Table 1:** Demographics data.

**Patient Population**	**Number**	**%**
Total no.	39	100
Died	0	0
Available	39	100
**Sex**	**Number**	**%**
Male	23	59
Female	16	41
**Age**	**Average (Y)**	**SD**
	65.9	11.8
**BMI**	**Average (kg/m^2^)**	**SD**
	25.3	5.47
**Side**	**Number**	**%**
Left	27	69.2
Right	12	30.8
**Comorbidity**	**Number**	**%**
Diabetes mellitus	5	12.8
Active smoking	3	7.7
Rheumatoid arthritis	2	5.1
Immunodeficiency	2	5.1
**Primary arthroplasty**	**Number**	**%**
THA	11	28.2
TKA	21	53.8
UKA	7	18
**Acute PJI**	**Number**	**%**
Early post-operative	31	79.5
Hematogenous	8	20.5

**Table 2:** Pre-operative criteria for the diagnosis of PJI according to the MSIS ICM 2018 (Erythrocyte Sedimentation Rate, ESR; C-Reactive Protein, CRP; White Blood Cells, WBC; Polymorphonuclear Neutrophils, PMN).

Criteria	Number	%
Sinus tract	3	7.7
Elevated serum CRP	39	100
Elevated ESR	34	87.2
Elevated Synovial WBC	36	92.3
Positive Alpha-defensin	33	84.6
Elevated Synovial PMN %	36	92.3
Elevated Synovial CRP	38	97.4

**Table 3:** Intra-operative criteria for the diagnosis of PJI according to the MSIS ICM 2018 and surgical time.

**Intraoperative Criteria**	**Number**	**%**
Positive Histology	3	7.7
Positive Purulence	39	100
**Positive colture**	**Number**	**%**
	37	94.9
**Surgical time**	**Average (min)**	**SD**
	79.9	17.9

## Data Availability

The data presented in this study are available on request from the corresponding author (privacy).
